# Human Recombinant Hyaluronidase Injections For Upper Limb Muscle Stiffness in Individuals With Cerebral Injury: A Case Series

**DOI:** 10.1016/j.ebiom.2016.05.014

**Published:** 2016-05-13

**Authors:** Preeti Raghavan, Ying Lu, Mona Mirchandani, Antonio Stecco

**Affiliations:** a Rusk Rehabilitation, New York University School of Medicine, New York University, New York, United States; b Steinhardt School of Education, Culture and Human Development, New York University, New York, United States; c University of Padua, Padua, Italy

**Keywords:** Connective tissue, Stroke, Hypertonia, Fascia, Spasticity, Hyaluronidase, Motor control, Case series

## Abstract

Spasticity, muscle stiffness and contracture cause severe disability after central nervous system injury. However, current treatment options for spasticity produce muscle weakness which can impede movement, and do not directly address muscle stiffness. Here we propose that the accumulation of hyaluronan within muscles promotes the development of muscle stiffness, and report that treatment with the enzyme hyaluronidase increases upper limb movement and reduces muscle stiffness without producing weakness. 20 patients with unilateral upper limb spasticity received multiple intramuscular injections of human recombinant hyaluronidase with saline at a single visit. The safety and efficacy of the injections, passive and active movement, and muscle stiffness at eight upper limb joints were assessed at four time points: pre-injection (T0), within 2 weeks (T1), within 4–6 weeks (T2), and within 3–5 months post-injection (T3). There were no clinically significant adverse effects from the injections. Passive movement at all joints, and active movement at most joints increased at T1, and persisted at T2 and T3 for most joints. The modified Ashworth scores also declined significantly over time post-injection. Hyaluronidase injections offer a safe and potentially efficacious treatment for muscle stiffness in neurologically impaired individuals. These results warrant confirmation in placebo-controlled clinical trials.

## Highlights

• The accumulation of hyaluronan within muscles contributes to the development of muscle stiffness after neurologic injury.
• Intramuscular injection of hyaluronidase reduces muscle stiffness and increases passive and active movement.
• The injections did not produce weakness or clinically significant adverse effects in the reported case series.

Muscle stiffness contributes to disability after neurologic injury. However both the cause of the muscle stiffness and its treatment remain to be established. We propose that the accumulation of hyaluronan within muscles promotes the development of muscle stiffness. We report that the enzyme hyaluronidase, which hydrolyzes hyaluronan, reduces muscle stiffness and increases both passive and active movement in individuals with spastic paralysis after cerebral injury. The treatment takes effect within days to 2 weeks, lasts for at least three months, does not produce weakness, and is safe without clinically significant adverse effects. These results present a promising treatment for a widespread problem.

## 1 Introduction

Spasticity is a common movement disorder after neurologic injury of cerebral and spinal origin such as stroke, traumatic brain injury, brain tumor, cerebral palsy, spinal cord injury, and multiple sclerosis. Upper limb spasticity is associated with reduced functional independence and a four-fold increase in direct care costs during the first year post-stroke alone (Lundstrom et al., 2010). The prevalence of spasticity increases over time, contributing to further disability long after the neurologic injury (Lundstrom et al., 2008). Spasticity is challenging to treat because the underlying neural and non-neural mechanisms and their interactions are not fully understood.

The neural mechanism underlying spasticity is hyper-excitability of the stretch reflex due to disinhibition of cortical influences on spinal cord circuitry, which results in velocity-dependent increase in tonic stretch reflexes (Lance, 1980). However, many patients with spasticity do not show any signs of hyperreflexia (Sinkjaer and Magnussen, 1994). Instead, muscle stiffness, defined here as increased resistance to passive movement, is the most common presenting sign in individuals with spasticity (Sheean and McGuire, 2009). Muscle stiffness adds further insult to the underlying weakness. It both prevents full passive movement (leading to abnormal posturing that can become fixed over time) and makes active movement more difficult in patients who are already weak from the neurologic injury. Non-neural peripheral mechanisms are thought to cause muscle stiffness (Burke et al., 2013, Stecco et al., 2014), although this has not been shown conclusively.

Current treatment options for spasticity include oral medications such as benzodiazepines, baclofen, and tizanidine that are central nervous system depressants used to suppress spinal hyper-excitability, and local injections of botulinum toxin used to suppress muscle over-activity. Whereas the oral medications can produce cognitive deficits, fatigue, and muscle weakness, botulinum toxin injections produce focal muscle weakness (Phadke et al., 2015). It is thus necessary to carefully balance the risks and benefits of treatment, which often remains inadequate. Furthermore, these treatments do not directly address muscle stiffness.

Here, we propose the Hyaluronan Hypothesis, which postulates that the accumulation of hyaluronan within muscles promotes the development of muscle stiffness. We report that the enzyme hyaluronidase, which hydrolyzes hyaluronan, and is available for off-label clinical use, increases both passive and active joint movement, and reduces muscle stiffness in individuals with upper limb spasticity. These results fill a critical gap in the understanding of muscle stiffness, and present a promising treatment for a vexing and widespread problem.

### 1.1 The Hyaluronan Hypothesis

Paralysis and immobility from central nervous system injury leads to rapid atrophy of muscle fibers (Springer et al., 2014), with a relative increase in the proportion of extracellular matrix, particularly in the perimysium surrounding the neurovascular tissues (de Bruin et al., 2014). Hyaluronan, a non-sulfated high molecular weight glycosaminoglycan, is particularly abundant around the endomysium, perimysium and epimysium of muscles (Piehl-Aulin et al., 1985, Laurent et al., 1991), where it provides lubrication to facilitate sliding and myofascial force transmission within and between muscles (Huijing and Jaspers, 2005). The concentration of hyaluronan can increase in serum, due to increased production after cerebral injury (Al'Qteishat et al., 2006), and in muscles from immobility (Okita et al., 2004), due to increased production, and/or decreased degradation (Jenkins et al., 2004). At high concentrations, hyaluronan itself, as well as protein-crosslinked networks or fibrillar assemblies of hyaluronan, can dramatically increase the viscoelasticity of the extracellular matrix (Matteini et al., 2009, Cowman et al., 2015); this decreases sliding of muscle fibers and reduces force transmission (Purslow, 2010), leading to muscle shortening.

The shortening may predominate in muscles with large myofascial expansions (Stecco, 2015) such as the pectoralis major, the biceps brachii, and pronator teres, connecting them together and leading to posturing of the upper limb in a typical flexor synergy pattern characterized by shoulder internal rotation, elbow flexion, and forearm pronation. Untreated and unchecked, this abnormal chronic posturing can lead to fibrosis and contracture. In fact, the accumulation of hyaluronan signals fibrosis (Jenkins et al., 2004). The aggregation of hyaluronan can disturb the balance of forces between the agonist and antagonist muscles leading to co-contraction, muscle fatigue, and excessive torques during movement (Hufschmidt and Mauritz, 1985). Thus the Hyaluronan Hypothesis provides a biomechanical explanation of how non-neural factors contribute to the development of muscle stiffness.

To test the Hyaluronan Hypothesis, we used the enzyme hyaluronidase to hydrolyze hyaluronan, reduce its molecular weight, and lower the viscosity of the extracellular matrix (Cowman et al., 2015). Hyaluronidase modifies the permeability of connective tissue through the hydrolysis of hyaluronan by splitting the glucosaminidic bond between C1 of an *N*-acetylglucosamine moiety and C4 of a glucuronic acid moiety. This temporarily decreases the viscosity of the extracellular matrix. The purpose of this case series is to describe the safety, tolerability, and preliminary efficacy of intramuscular injections of off-label human recombinant hyaluronidase-saline injections in increasing passive and active joint movement and reducing upper limb muscle stiffness in patients with cerebral injury. Human recombinant hyaluronidase has been FDA-approved since 2005 as a tissue permeability modifier. It is currently indicated as an adjuvant in subcutaneous fluid administration for achieving hydration, to increase the dispersion and absorption of other injected drugs, such as cancer chemotherapeutics, and in subcutaneous urography for improving resorption of radiopaque agents.

We present evidence that intramuscular injections of off-label human recombinant hyaluronidase offer a safe and potentially efficacious treatment for muscle stiffness that can increase passive and active joint movement, without producing weakness, in individuals with neurologic injury.

## 2 Methods

### 2.1 Patients

From May 2014 to September 2015, twenty patients (13 male and 7 female) between 10–77 years with moderately severe upper limb muscle stiffness in more than one joint, consented to and received off-label injections of recombinant hyaluronidase in combination with preservative-free normal saline, in the outpatient hand clinic at the Hospital for Joint Diseases, New York University Langone Medical Center. The patients had exhausted all available options with limited benefit, or were referred by providers seeking this specific off-label treatment. The institutional review board approved the case series (# i15-00333). All procedures were performed in accordance with the Guidelines for Good Clinical Practice as issued by the International Conference on Harmonization of Technical Requirements for Registration of Pharmaceuticals for Human Use. The investigation and the use of patient data for research purposes were in accordance with the Declaration of the World Medical Association, and the study was performed in accordance with ethical standards on human experimentation as per the Helsinki Declaration.

The inclusion criteria for the present case series were: informed consent for the procedure and for documentation of video recordings of joint movement for clinical and academic purposes, and moderately severe muscle stiffness in more than one joint of a single upper limb defined by a modified Ashworth scale (MAS) score of ≥ 2. The modified Ashworth scale measures resistance to passive movement and is a common clinically used measure of muscle stiffness rather than spasticity. Exclusion criteria were severe sensory aphasia, bilateral upper limb weakness precluding compliance with a home exercise program using the unaffected limb to mobilize the affected limb, concurrent treatment of spasticity with other injectable agents such as botulinum toxin injections, and recent changes in the treatment of spasticity or underlying medical problems. Initial suitability for the injections was assessed by obtaining a history of known hypersensitivity to eggs or vaccines produced in the same manner as human recombinant hyaluronidase. The 20 cases are described in Table 1.

### 2.2 Injection With Human Recombinant Hyaluronidase

Hyaluronidase is supplied as a sterile, clear, colorless, non-preserved, ready-for-use solution. Each mL contains 150 USP units of recombinant human hyaluronidase with 8.5 mg sodium chloride, 1.4 mg dibasic sodium phosphate, 1 mg albumin human, 1.5 mg l-methionine, 0.2 mg polysorbate 80, and hydrochloric acid and sodium hydroxide added for pH adjustment; it has a pH of ~ 7.0 and an osmolality of 280–340 mOsm/kg. Given that hyaluronidase is known to be antigenic, a preliminary skin test for hypersensitivity to human recombinant hyaluronidase was performed. An intradermal injection of approximately 0.02 mL (3 units) of a 150 units/mL solution was injected. No erythema, itching, or wheal was noted in or around the injection site at 5 or 20 min in any of the patient's injected. There are no reported contraindications to intramuscular injection of hyaluronidase provided routine precautions to avoid intravascular injections are followed (Moore, 1950). The use of intramuscular procaine and hyaluronidase was previously reported for the treatment for spastic flatfoot (Locke, 1952). The dose of intramuscular hyaluronidase was determined according to each patient's pattern and extent of muscle stiffness, but the maximum dose used in this cohort was 600 IU, with no > 75 IU injected into a single site. The dosage selected is well below the threshold of toxicity of hyaluronidase (Seifter, 1950).

**Fig. 1:**
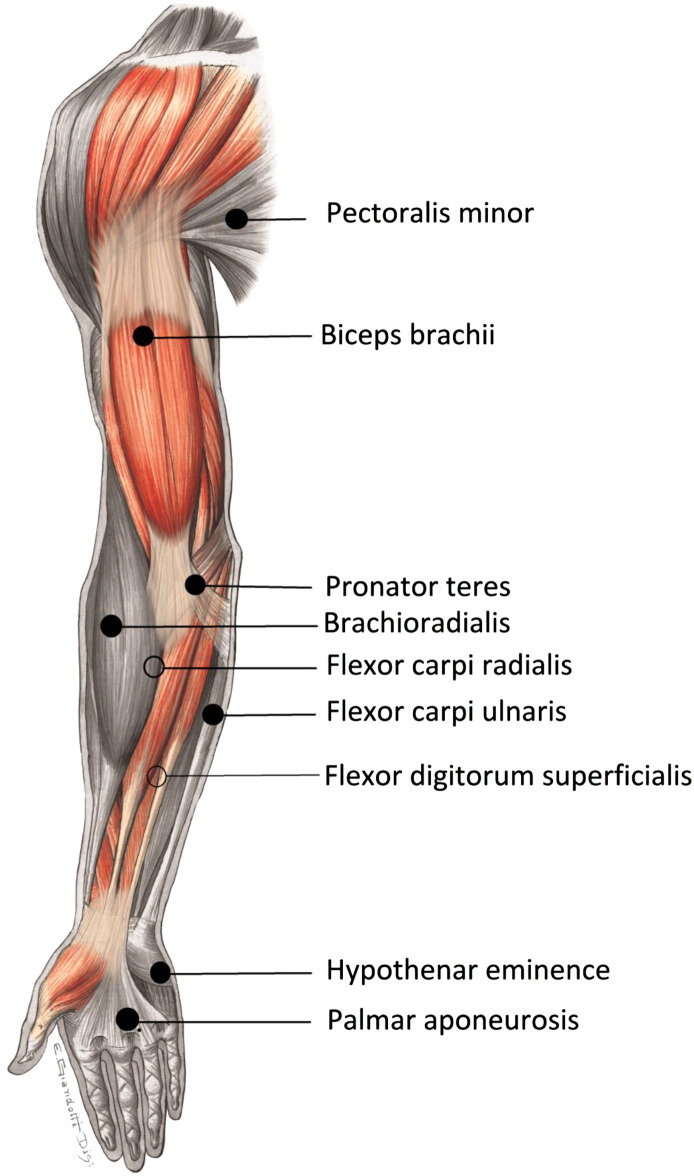
Muscles injected with Human Recombinant Hyaluronidase. Synergistically acting muscles contributing to the stiffness along the myofascial chain of the upper limb were selected for injection with Hylauronidase mixed with normal saline in a 1:1 ratio.

Hylauronidase was mixed with saline in a 1:1 ratio, with 1 mL (150 IU) of hyaluronidase diluted with 1 mL of normal saline. The typical recommended dose of hyaluronidase is 150 IU. We chose to dilute it further because the modification in tissue permeability induced by the administration of hyaluronidase is influenced not only by enzyme concentration, but also by the volume and pressure of the injection (Hechter, 1947). Dilution also resulted in a lower dosage overall across all the muscles injected. The dilution was constant across all patients and a 0.7 mm 30G needle was used for all the injections. The skin was cleaned with antiseptic swabs, and the injections were administered using sterile aseptic technique by the same operator (PR). Surface anatomical landmarks were used to locate the muscles injected and precautions were taken to avoid intravascular injection. Synergistically acting muscles in one or more spatial planes that appeared to contribute most to the stiffness along the myofascial chain of the upper limb (Stecco, 2015) were selected for injection. Fig. 1 shows the most common muscles injected across all patients. Patients were advised to use a warm compress for local soreness around the injections sites. Given that immobility is hypothesized to contribute to the alteration in muscle stiffness, the patients were encouraged to continue current therapy, and were prescribed a home exercise program of stretching and passive movements for the affected upper limb (Table 2).

### 2.3 Assessments

Patients underwent a thorough neurologic and musculoskeletal exam to document the extent of passive and active movement restriction and muscle stiffness in the affected upper limb at four time-points: pre-injection (T0), and post-injection within 2 weeks (T1), between 4–6 weeks (T2) and within 3–5 months (T3). Patients #1–6 (Table 1) were closely monitored within 1–3 days after the injections to examine the time of onset of therapeutic effect, if any. Safety was assessed by examination for adverse events and clinically significant changes in vital signs (body temperature, diastolic and systolic pressure, heart rate) immediately post-injection and over the period of follow-up. Adverse events were sought through interviews, during which patients were encouraged to report any problems. Each patient was assessed by the same physician throughout the course of treatment. All complaints were also assessed by an unbiased clinic nurse and documented in the medical record.

Passive and active movements were measured from video recordings of eight joint movements: shoulder abduction and forearm pronation and supination (in the frontal plane), and shoulder forward flexion, elbow flexion and extension, and wrist flexion and extension (in the sagittal plane), taken at each visit. The movements were recorded with the camera facing perpendicular to the plane of the movement. Each movement was repeated three times passively, and when possible, actively. For the first six patients who had little ability to move actively (#1–3, 5, and 6), we did not anticipate any changes in active movement; therefore these were not recorded. Finger and hand movements were recorded for many patients, but these could not be analyzed consistently. For examples, see videos of passive and active movements (Video 1, Video 2, Video 3, Video 4, Video 5, submitted with informed consent from patients). Joint excursions were extracted from the videos using commercially available video analysis software (Dartfish 7.0), which enables the measurement of angular movements digitally for quantitative clinical movement analysis. It is found to have moderate to excellent reliability for measurement of upper limb movement amplitude (interclass correlation coefficient, ICC = 0.98–0.99), (Melton et al., 2011) and inter-rater reliability for functional upper limb movement (ICC = 0.68–1.00) (Khadilkar et al., 2014). We have further validated this method with 3-dimensional motion analysis and conventional goniometry in the clinical research laboratory (unpublished data). However, the quality of the movements, the degree of compensation when isolated movements are not possible, and the angle of the camera can produce errors in determination of the excursions, particularly for forearm pronation and supination. The technique for measurement of forearm pronation and supination using a camera placed in front of the forearm tended to overestimate the angle of excursion, which were therefore capped at 100°. To control for measurement errors and inadvertent bias, the best of the three trials performed was evaluated. Also, two authors (AS and MM) independently extracted the joint excursions from the same videos and the degree of correlation between their measurements was assessed.

Muscle stiffness, defined as the resistance to passive movement, was assessed with the modified Ashworth scale (MAS) at each time point. The modified Ashworth scores do not take the velocity dependency of the resistance to passive movement into account, and it is recommended that this scale should no longer be used to measure spasticity (Fleuren et al., 2010). However, in the clinical setting for this case series, the Ashworth scores were a useful tool to measure muscle stiffness. The scores were recorded in the medical chart where they were extracted by one author who was not involved in the care of the patients (MM).

### 2.4 Statistical Analysis

Data analysis was performed using Rstudio (version 0.99). Due to the small sample size, the ordinal nature of the modified Ashworth scale, and to avoid violation of normality assumption, nonparametric tests were used for inference for the three outcome measures. The Wilcoxon-Mann-Whitney test was used to assess changes in short-term and long-term outcomes post-injection. Hommel's adjusted p-values were used to control for multiple hypothesis testing (Hommel, 1988). Spearman and Kendall tests were used to assess the correlation between pre-injection measurements, dosage, age, gender, time since neurological injury, compliance with home exercise program, short-term (T1–T0) and long-term (T3–T0) change in passive and active movement, and modified Ashworth scores. Mean and standard deviation are reported for the continuous data (passive and active movement) and median and interquartile range, and proportion of scores is reported for the ordinal data (modified Ashworth scores). To control for investigator bias in extraction of joint movement from the videos, two raters independently measured the range of motion for each subject at every joint. Given that the joint movements independently extracted by the two investigators were highly correlated (Pearson's correlation coefficient = 0.98, weighted Kappa coefficient = 0.97), their average was used in the statistical analyses. To further control investigator bias and manage conflict of interest, the findings were reviewed by an independent third-party with expertise in the field who is not associated with New York University.

### 2.5 Role of the Funding Source

No funding or drug was provided by any pharmaceutical company for this case series. The drug was obtained from the hospital pharmacy for off-label clinical use. The corresponding author had full access to all the data in this study and had final responsibility for the decision to submit for publication.

## 3 Results

All patients (n = 20) were assessed pre-injection (T0) and within 2 weeks post-injection (T1). The mean time for post-injection assessments was 7 (SD 4) days. All patients tolerated the injections without immediate adverse effects. None of the patients demonstrated a hypersensitivity reaction to the skin test. The most common adverse reaction was soreness at the injection sites with onset within 24 h of the injection, lasting 24–48 h. The mean time for first post-injection follow up (T2) was 43 (SD 15) days, and it was 96 (SD 39) days for the second post-injection follow up (T3). Three patients (#13, 17, and 19) did not present for follow up within the T2 and T3 time frame. Patients #13 and 17 did not experience any adverse effects. Patient #19 experienced a delayed hypersensitivity reaction characterized by a pruritic rash over the injected areas on post-injection day two, which subsided on treatment with oral diphenhydramine and topical 1% hydrocortisone over 7 days. Four patients (#1, 2, 4, and 7) did not present for follow-up within the T3 time frame. Patient #1 developed an unrelated wound on his back from a burn, patient #2 did not experience any adverse effects, patient #4 had an unrelated fall with a humeral fracture, and patient #7 had an unrelated fall from a treadmill. The patients subsequently healed and were followed in the clinic. Of note, 15 of the 20 patients (Table 1) voluntarily received subsequent treatment with hyaluronidase after the period of follow-up specified in this report.

The passive movement increased significantly at all joints at T1, and persisted at T2 and T3 for all joints, except for elbow flexion and forearm supination (Table 3). Note that the passive range of motion for elbow extension and forearm supination for most of the patients was already within 10% of the maximum possible range at T0 (see Appendix A for figures of individual subject data). For elbow extension the change in passive movement was negatively correlated with the pre-injection movement; the greater the pre-injection elbow extension, the smaller its increase post-injection in both the short-term (r = − 0.86, p < 0.001) and long-term (r = − 0.70, p = 0.025). However, for forearm supination the opposite was seen; the more restricted the forearm supination pre-injection at T0, the smaller its increase post-injection in the short-term (r = 0.57, p = 0.03) (Table 4).

Active movement showed more variability across patients. For patients who could perform active movements, the mean extent of movement increased significantly at all joints in the short-term, except for elbow extension, forearm pronation, and wrist extension (Table 3). However, over the long-term, the increase was significant at all joints except wrist extension. The change in active movement was negatively correlated with the pre-injection movement; the more restricted the pre-injection movement the greater its increase post-injection in the short-term for elbow flexion (r = − 0.62, p = 0.004), and in the long-term for elbow flexion (r = − 0.58, p = 0.04), forearm pronation (r = − 0.65, p = 0.017), and wrist flexion (r = − 0.68, p = 0.011) (Table 4).

The median modified Ashworth scores decreased significantly across all the joint movements from pre- to post-injection assessments (Table 3). The change in stiffness was negatively correlated with the degree of pre-injection stiffness. The greater the pre-injection scores, the smaller the change for most movements (except shoulder flexion and wrist extension) in the short-term, and for shoulder abduction (r = − 0.84, p < 0.001), elbow extension (r = − 0.85, p < 0.001), and forearm supination (r = − 0.67, p = 0.013) in the long-term (Table 4). Table 5 shows that the modified Ashworth scores across all movements and subjects reduced from a score of 2 (51%) and 3 (44%) at T0 to mostly 0 (48%) and 1 (30%) at T1.

Further correlation analyses showed that neither the short-term nor the long-term changes in passive or active movement, or modified Ashworth scores were significantly correlated with patients' characteristics such as age, sex, side of hemiparesis, etiology, time since injury, prior treatment, absolute or weight-adjusted dose of hyaluronidase, number of sites injected, or compliance with the exercise program.

## 4 Discussion

In this case series, we report that intramuscular injections of the enzyme hyaluronidase increased passive and active joint movement and reduced muscle stiffness at upper limb joints in patients with spasticity of cerebral origin. The effect of treatment remained over at least three months of follow-up. These results suggest that accumulation of hyaluronan within muscles promotes the development of muscle stiffness in individuals with neurologic injury, and that intramuscular delivery of hyaluronidase is a promising direct treatment for muscle stiffness. The injections were safe and well tolerated, and without clinically significant adverse effects. Most importantly, the treatment did not produce weakness, which is a common adverse effect with current treatment options for spasticity (Phadke et al., 2015). Side effects of muscle weakness and fatigue are major impediments to recovery in patients with neurologic injury. The ability of hyaluronidase to reduce the resistance to movement without producing weakness suggests that it can potentially facilitate recovery of function after neurologic injury.

There is no direct evidence yet that the change in mechanical properties of spastic muscles is due to the accumulation of hyaluronan. However, a study by Okita et al. (2004) showed that immobilization of the rat ankle joint led to increased hyaluronan accumulation in the endomysium of the rat soleus muscle compared to controls within 1 week, with concomitant decrease in sarcomere length. This was followed by subsequent change in the arrangement of collagen fibrils and reduction in ankle range of motion. The authors suggested that increased hyaluronan in muscular tissue may induce muscle stiffness. There is now emerging evidence that the accumulation of hyaluronan precedes fibrosis in several organs including the lung, kidney, and liver (Bensadoun et al., 1996, Evanko et al., 2015, Jun et al., 1997, Hernnas et al., 1992, Halfon et al., 2005). Although the mechanisms underlying changes in muscle due to mechanical immobilization and spastic paralysis may be different, immobility is a consequence of spastic paralysis, and may trigger a cascade of events that leads to the accumulation of hyaluronan (Stecco et al., 2014).

Furthermore, there is evidence for the role of transforming growth factor-β (TGF-β), a profibrotic cytokine, in the regulation of hyaluronan production (Heldin et al., 1989, Westergren-Thorsson et al., 1990, Dubaybo and Thet, 1990). Tissue injury, including cerebral injury, leads to significant increase in TGF-β locally (Logan et al., 1992, Wang et al., 1995), where it may play a role in anti-inflammatory processes and in brain tissue remodeling, as well as in the serum (Kaestner and Dimitriou, 2013, Shi et al., 2015), where it may trigger catabolic signaling for muscle atrophy (Springer et al., 2014, Budasz-Rwiderska et al., 2005), stimulate the production of hyaluronan (Al'Qteishat et al., 2006), and the migration (Samuel et al., 1993) and differentiation of myofibroblasts leading to fibrosis (Webber et al., 2009a, Webber et al., 2009b). Moreover, catabolism of hyaluronan by hyaluronidases (Colombaro et al., 2015), and TGF-β antagonism (Midgley et al., 2015) can reduce the levels of accumulated hyaluronan, potentially disrupting progression to fibrosis. Taken together, these findings support the Hyaluronan Hypothesis of muscle stiffness and the treatment of muscle stiffness with hyaluronidase.

Hyaluronidase is FDA-approved as an agent that enhances tissue permeability, and it is currently indicated as an adjuvant to facilitate the absorption of drugs. The action of hyaluronidase is short-lived; the dermal barrier to fluid diffusion is partly restored in 24 h and completely restored in 48 h following the administration of hyaluronidase (Hechter, 1948). However, our results show noticeable and persistent reduction in muscle stiffness after 48 h; this may suggest the presence of hyaluronan cables (de la Motte et al., 2003, Lauer et al., 2013, Zhao et al., 1995) in muscle stiffness that degrade slowly, and/or possible cross-reactivity with chondroitinase (Stern and Jedrzejas, 2006). Bacterial chondroitinase degrades scar tissue and promotes neural plasticity after central nervous system damage in animal models (Fawcett, 2015). Hence longer-term results with hyaluronidase injections may suggest interactions between non-neural muscle stiffness and neural plasticity.

The efficacy of treatment with hyaluronidase may be limited by the degree of baseline stiffness, fibrosis, and capacity for movement in specific directions. Increased stiffness in inter-connected synergistic muscles may contribute to the flexor synergy pattern, and chronic abnormal posturing in shoulder adduction, elbow flexion, and forearm pronation is frequently seen in patients with spastic hemiparesis. Some degree of fibrosis in these muscles may limit the efficacy of treatment with hyaluronidase and explain the negative correlation between baseline and long-term change scores on the Modified Ashworth scale for shoulder abduction, elbow extension and forearm supination, and the positive correlation with change in short-term passive forearm supination. The negative correlation between baseline and change in passive elbow extension over the short- and long-term suggest ceiling effects as most of the patients had almost full passive range at the elbow at baseline. The negative correlations between baseline and change in active elbow flexion, forearm pronation, and wrist flexion suggest greater capacity for movement in these directions.

Furthermore, stretching a stiff muscle either passively or actively (for example, by contraction of the antagonist), will restrain the movement due to increased tissue stiffness, and restrain the stretch due to increased reflex contractions as a result of increased sensitivity of the muscle spindles embedded in stiff tissue. Increased reflex contractions may stimulate further production of hyaluronan (Stecco et al., 2011) increasing connective tissue viscoelasticity and muscle stiffness. Since attempts to stretch a muscle will invariable stretch multiple interconnected synergist and antagonist muscles (Stecco, 2015), reduction of stiffness following restoration of intramuscular viscosity may not persist without concurrent treatment of several muscles. In the first few patients we injected relatively few muscles, and subsequently increased the number of muscles injected as we learnt that injecting muscles both proximal and distal to the primary stiff muscle, along the agonist and antagonist myofascial chains of limb movement, can give more favorable results. An understanding of fascial planes and barriers to diffusion of hyaluronidase, and a comprehensive and accurate selection of muscles for injection are therefore important aspects of the treatment. Use of tools such as ultrasonography or other imaging techniques to assess the extent of fibrosis could improve patient and muscle selection for treatment.

So far, hyaluronidase has only been used for the treatment of muscle stiffness at our clinic in New York. However, there are no technical limitations that would prevent this procedure from being performed in other centers with similar expertise. The drug is available for off-label use in the United States. A blinded randomized placebo-controlled trial will be needed to control for a placebo response, the effects of confounding variables such as number and location of injection sites, amount and quality of therapy, and investigator bias in recording and analyzing movement. This report does not address the effect of repeated drug administration, although 15/20 patients did return for further injections. The results of this study warrant replication by independent groups.

Future studies should also directly investigate the role of hyaluronan and hyaluronidase in changing the mechanical properties of spastic muscles using simultaneous joint angle, torque, and EMG measurements to tease apart the effects on spasticity versus stiffness, and examine the efficacy of treatment with hyaluronidase on improvement in motor control, prevention of muscle contracture, reduction in sensorimotor impairment, and increase in quality of life. In addition the role of cytokines such as TGF-β after cerebral injury in mediating sarcopenia, accumulation of hyaluronan in muscle, and muscle contracture warrants further study.

This case series provides preliminary evidence for the safety and potential efficacy of hyaluronidase injections as a treatment for muscle stiffness that may enhance functional recovery in the spastic upper limb, and may be applicable to other disorders characterized by muscle stiffness.

The following are the supplementary data related to this article.

Video 1 Case video of active elbow flexion-extension before and after hyaluronidase injections. Video 1

Video 2 Case video of wrist and finger extension before and after hyaluronidase injections. Video 2

Video 3 Case video of passive shoulder abduction before and after hyaluronidase injections. Video 3

Video 4 Case video of active forearm pronation and supination before and after hyaluronidase injections. Video 4

Video 5 Case video of passive shoulder flexion before and after hyaluronidase injections. Video 5

Appendix A Individual subject passive and active range of motion data extracted independently by two investigators. Image 1

## Contributors

PR and AS conceived of the idea. PR performed the injections, recorded data, interpreted the data, and wrote the report. MM extracted clinical data from medical records and movement data from videos, and revised the report. YL performed statistical analysis, interpreted the data and wrote the report. AS extracted movement data from videos and wrote the report.

## Declaration of Interest

PR and AS report that New York University has filed a patent (PCT/US 15/40767) on hyaluronidase for muscle stiffness. AS is the president of the Fascial Manipulation Association, a non-profit organization whose purpose is to spread information about fascia and promote research on fascia. PR is a co-founder of Mirrored Motion Works, Inc., a company that creates devices and services for rehabilitation, and serves as the Chairperson of YoungStroke, Inc., a non-profit organization dedicated to advocacy for young adult stroke survivors. MM and YL declare no competing interests.

## Figures and Tables

**Table 1:** Characteristics of patients.

Case #	Age (years)	Sex	Side of hemiparesis	Etiology	Time since injury (months)	Prior treatment	Dose injected (IU)/# sites	Dose per weight IU/kg	Muscles injected	Compliance with exercise program
1	67	Male	Right	Ischemic stroke	29	Botulinum Toxin	450/6	5.09	Pectoralis minor, triceps, pronator teres, flexor carpi radialis, extensor carpi ulnaris, extensor digitorum communis	Non-compliant
2	48	Female	Right	Hemorrhagic stroke	64	Botulinum Toxin	450/6	8.65	Pectoralis minor, pronator teres, flexor carpi radialis, extensor carpi ulnaris, flexor digitorum, extensor digitorum communis	Non-compliant
3	59	Male	Left	Ischemic stroke	61	Botulinum Toxin	450/6	5.51	Pectoralis minor, pronator teres, flexor carpi radialis, extensor carpi ulnaris, flexor digitorum, extensor digitorum communis	Compliant
4^a^	53	Female	Left	Hemorrhagic stroke	54	Botulinum Toxin	450/7	5.10	Pectoralis minor, biceps brachi, pronator teres, flexor carpi radialis, extensor carpi ulnaris, extensor digitorum communis, abductor pollicis	Non-compliant
5^a^	66	Male	Right	Hemorrhagic stroke	41	Botulinum Toxin	450/6	4.10	Pectoralis minor, biceps brachi, pronator teres, flexor carpi radialis, extensor carpi ulnaris, extensor digitorum communis	Non-compliant
6	43	Female	Left	Ischemic stroke	45	None	450/6	7.87	Pectoralis minor, brachialis, pronator teres, flexor digitorum, extensor digitorum communis, palmar aponeurosis	Compliant
7	43	Male	Right	Ischemic stroke	10	Botulinum Toxin	600/8	7.08	Pectoralis minor, teres major, pronator teres, flexor carpi radialis, extensor carpi ulnaris, flexor digitorum, extensor digitorum communis, palmar aponeurosis	Compliant
8	59	Male	Right	Ischemic stroke	24	Botulinum Toxin	600/8	6.58	Pectoralis minor, teres major, pronator teres, flexor carpi radialis, extensor carpi ulnaris, extensor digitorum communis, flexor digitorum, palmar aponeurosis	Compliant
9^a^	64	Male	Right	Hemorrhagic stroke	85	None	600/8	7.16	Pectoralis minor, biceps brachi, pronator teres, flexor carpi radialis, extensor carpi ulnaris, flexor digitorum, extensor digitorum communis, palmar aponeurosis	Compliant
10	15	Male	Right	Ischemic stroke (carotid dissection)	5	None	600/8	10.24	Pectoralis minor, biceps brachi, brachialis, pronator teres, flexor carpi ulnaris, flexor digitorum, extensor digitorum communis, palmar aponeurosis	Compliant
11	11	Female	Left	Cerebral palsy	114	Botulinum Toxin	450/7	14.66	Pectoralis major, biceps brachi, pronator teres, flexor carpi ulnaris, extensor carpi radialis, flexor digitorum, palmar aponeurosis	Non-compliant
12	38	Female	Right	Ischemic stroke	18	None	600/8	9.84	Pectoralis minor, biceps brachi, brachialis, pronator teres, flexor carpi ulnaris, flexor digitorum, extensor digitorum communis, palmar aponeurosis	Compliant
13^a^	10	Male	Right	Cerebral palsy	152	Botulinum Toxin	300/6	5.55	Pectoralis minor, biceps brachi, flexor carpi radialis, flexor carpi ulnaris, flexor digitorum, extensor digitorum communis	Non-compliant
14	39	Male	Left	Hemorrhagic stroke	35	Botulinum Toxin	450/9	6.94	Pectoralis minor, biceps brachi, brachialis, pronator teres, flexor carpi ulnaris, extensor digitorum communis, palmar aponeurosis, interosseus III-IV and IV-V	Compliant
15	11	Male	Right	Brain tumor resection	8	None	450/9	10.54	Pectoralis minor, biceps brachi, flexor carpi ulnaris, flexor carpis radialis, flexor digitorum, extensor digitorum communis, palmaris aponeurosis, interosseus III-IV and IV-V	Compliant
16	10	Female	Right	Stroke (AVM rupture)	17	None	300/5	10.38	Pronator teres, brachialis, extensor carpi ulnaris, flexor digitorum, hypothenar eminence	Compliant
17	15	Female	Right	Brain tumor resection	13	Botulinum Toxin	600/9	9.48	Levator scapula, pectoralis minor, biceps brachi, brachioradialis, pronator teres, flexor carpi ulnaris, extensor carpi radialis, flexor digitorum, extensor digitorum communis, palmar aponeurosis	Compliant
18	58	Male	Right	Hemorrhagic stroke	12	None	600/8	8.10	Romboid minor, pectoralis minor, biceps brachi, brachialis, pronator teres, flexor carpi ulnaris, flexor digitorum, extensor digitorum communis	Non-compliant
19^a^	77	Male	Left	Ischemic stroke	19	None	450/5	4.72	Posterior deltoid, pectoralis minor, pronator teres, flexor carpi radialis, extensor digitorum communis	Compliant
20	37	Male	Right	Radiation-induced vasculitis	7	Botulinum Toxin	600/9	8.82	Levator scapula, pectoralis minor, brachialis, pronator teres, flexor carpi ulnaris, flexor digitorum, extensor digitorum communis, abductor pollicis, palmar aponeurosis	Compliant

a Patients that did not receive subsequent hyaluronidase injections.

**Table 2:** Home exercise program.

Joint	Exercise	# Repetitions
Shoulder flexion-extension	While lying down hold the pipe in both hands, raise arms up above the head and then down	20
While seated hold the pipe in both hands, raise arms up above the head and then down	20
Elbow flexion-extension	While seated hold the pipe in both hands, bring arm outward from the chest and then inward	20
Forearm pronation-supination	Hold pipe from underneath with unaffected hand, and twist the affected forearm to bring it palm up as far as it will go	20
Wrist flexion-extension	Fix the upper arm to the side of the body or place the forearms on an arm chair. Hold the pipe with both hands and raise the wrist up and down	20
Finger extension-abduction	Bring hands into prayer position with fingers of the unaffected hand opposing the same finger of the affected hand, separate the fingers repeatedly	20

All patients were prescribed a daily home-exercise program and compliance was documented in the medical chart. They were asked to hold a pipe (or stick), approximately shoulder width in length, with both hands and perform the movements listed. Here the unaffected arm guides and facilitates the movements of the affected arm.

**Table 3:** Mean (SD) passive and active movement and median (IQR) modified Ashworth score at each time point and significance of change.

	Shoulder flexion (max 180°)	Shoulder abduction (max 180°)	Elbow flexion (max 150°)	Elbow extension (max 180°)	Forearm pronation (max 100°)	Forearm supination (max 100°)	Wrist flexion (max 90°)	Wrist extension (max 90°)
Passive movement								
T0	132.85 (30.82)	131.3 (28.82)	142.3 (13.40)	173.35 (12.94)	88.4 (13.06)	82.3 (25.88)	72 (11.74)	47.2 (21.35)
T1	148.75 (26.95)	149.1 (27.94)	146.7 (11.66)	176.55 (10.06)	93.95 (8.40)	91.35 (13.13)	78.7 (9.66)	65.75 (22.62)
T1–T0 (n = 20) p-value	< 0.001	< 0.001	0.03	0.02	0.004	0.02	0.001	< 0.001
T2	149.88 (24.02)	150.65 (32.44)	147.35 (11.32)	177.82 (5.75)	93.76 (9.25)	91.18 (16.88)	78.82 (10.99)	62.47 (18.36)
T3	151.38 (29.04)	160.85 (22.59)	147.92 (14.44)	178.85 (3.0)	94.38 (12.04)	94.30 (11.43)	80.62 (12.22)	64 (21.59)
T3–T0 (n = 13) p-value	0.001	0.002	0.09	0.004	0.004	0.23	0.02	0.01

Active movement								
T0	101.8 (52.74)	98.53 (42.79)	129.33 (13.11)	158.87 (28.13)	34.6 (37.44)	35.2 (32.91)	25.93 (26.52)	21.53 (19.10)
T1	113.87 (55.41)	110.2 (40.94)	137.27 (13.39)	164.6 (21.88)	41.73 (40.23)	41.8 (36.25)	36.33 (28.29)	25.6 (20.68)
T1–T0 (n = 15) p-value	< 0.001	< 0.001	< 0.001	0.25	0.06	0.01	0.003	0.18
T2	106.33 (56.80)	104.5 (42.87)	138.67 (12.42)	164.83 (28.24)	36.58 (36.05)	42.17 (35.70)	37.75 (26.51)	26.67 (19.39)
T3	117.5 (52.37)	113.08 (39.81)	145.6 (11.42)	168.7 (24.69)	50.6 (37.18)	54.7 (31.10)	48.8 (28.15)	31.3 (19.10)
T3–T0 (n = 10) p-value	0.002	0.01	0.002	0.025	0.02	0.004	0.004	0.104

Modified Ashworth score								
T0	3 (1)	3 (1)	2 (1)	2 (1)	2 (1)	2 (1)	2 (1)	2.5 (1)
T1	1 (2)	1 (2)	0 (1)	1 (0)	0 (1)	0.5 (2)	0 (1)	1 (2)
T1–T0 (n = 20) p-value	< 0.001	< 0.001	< 0.001	< 0.001	< 0.001	< 0.001	< 0.001	< 0.001
T2	1 (1)	1 (2)	0 (0)	1 (1)	0 (1)	1 (1)	0 (0)	2 (1)
T3	0 (1)	0 (1)	0 (0)	1 (1)	0 (0)	0 (2)	0 (0)	1 (2)
T3–T0 (n = 13) p-value	< 0.001	< 0.001	< 0.001	< 0.001	< 0.001	0.001	< 0.001	0.005

T0 = pre-injection; T1 = within 2 weeks post-injection; T2 = within 6–8 weeks post-injection; T3 = within 3–5 months post-injection.

**Table 4:** Correlation between baseline and change scores.

	Passive movement		Active movement		Modified Ashworth scale	
Correlation coefficient	p-Value	Correlation coefficient	p-Value	Correlation coefficient	p-Value
Correlation between baseline and short-term change						
Shoulder flexion	0.26	0.35	− 0.13	0.58	− 0.37	0.11
Shoulder abduction	− 0.20	0.48	− 0.17	0.48	− 0.49	0.03
Elbow flexion	− 0.17	0.54	− 0.62	0.004	− 0.46	0.04
Elbow extension	− 0.86	< 0.001	− 0.19	0.42	− 0.87	< 0.001
Forearm pronation	0.43	0.11	− 0.13	0.60	− 0.75	< 0.001
Forearm supination	0.57	0.03	0.24	0.31	− 0.52	0.02
Wrist flexion	0.06	0.82	− 0.27	0.24	− 0.61	0.004
Wrist extension	0.19	0.49	0.08	0.75	− 0.35	0.13

Correlation between baseline and long-term change						
Shoulder flexion	0.20	0.58	− 0.40	0.18	− 0.48	0.09
Shoulder abduction	− 0.16	0.65	− 0.28	0.35	− 0.84	< 0.001
Elbow flexion	− 0.01	0.99	− 0.58	0.04	− 0.04	0.88
Elbow extension	− 0.70	0.03	− 0.18	0.55	− 0.85	< 0.001
Forearm pronation	− 0.18	0.62	− 0.65	0.017	− 0.47	0.10
Forearm supination	− 0.13	0.72	− 0.18	0.55	− 0.67	0.013
Wrist flexion	− 0.37	0.29	− 0.68	0.011	− 0.50	0.08
Wrist extension	− 0.18	0.63	− 0.22	0.47	− 0.39	0.19

Spearman's correlation for passive and active movement and modified Ashworth score between pre-injection baseline (T0) and short-term change (T1–T0), and baseline and long-term change (T3–T0).

**Table 5:** Change in distribution of modified Ashworth scale (MAS) scores over time.

Time	MAS = 0	MAS = 1	MAS = 1 +	MAS = 2	MAS = 3	Total no. of joints assessed
T0	2 (1.3%)	3 (1.9%)	3 (1.9%)	81 (50.6%)	71 (44.4%)	160
T1	66 (48.2%)	41 (29.9%)	1 (0.7%)	21 (15.3%)	8 (5.8%)	137
T2	62 (59%)	26 (24.8%)	1 (1%)	14 (13.3%)	2 (1.9%)	105
T3	72 (45%)	53 (33.1%)	2 (1.3%)	31 (19.4%)	2 (1.3%)	160

T0 = pre-injection; T1 = within 2 weeks post-injection; T2 = within 6–8 weeks post-injection; T3 = within 3–5 months post-injection.
