# Supplementary figures and images for: Human Recombinant Hyaluronidase Injections For Upper Limb Muscle Stiffness in Individuals With Cerebral Injury: A Case Series
Source: eBioMedicine. 2016 May 13;9:306–13. doi: 10.1016/j.ebiom.2016.05.014 (PMC4972484; doi:10.1016/j.ebiom.2016.05.014)

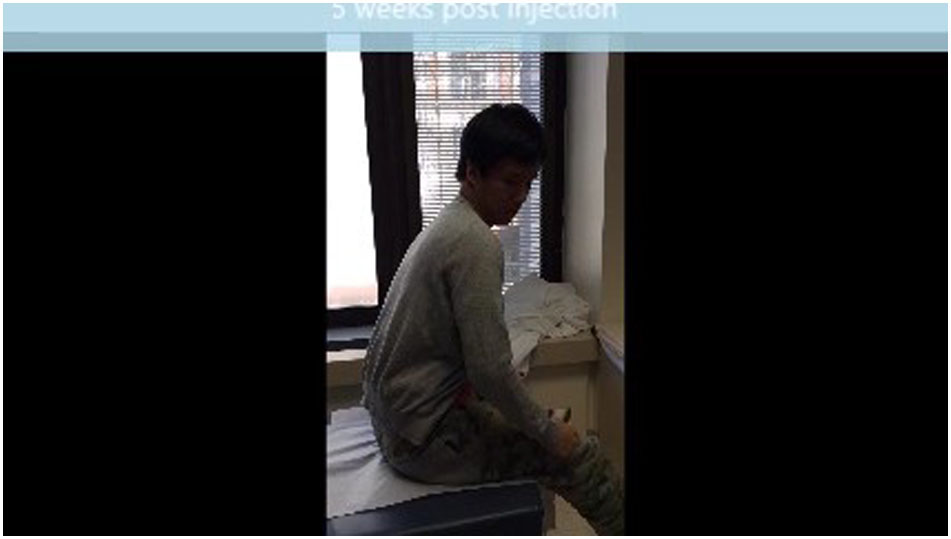

Supplement: Video 1 — Case video of active elbow flexion-extension before and after hyaluronidase injections. [file mmc1.jpg]

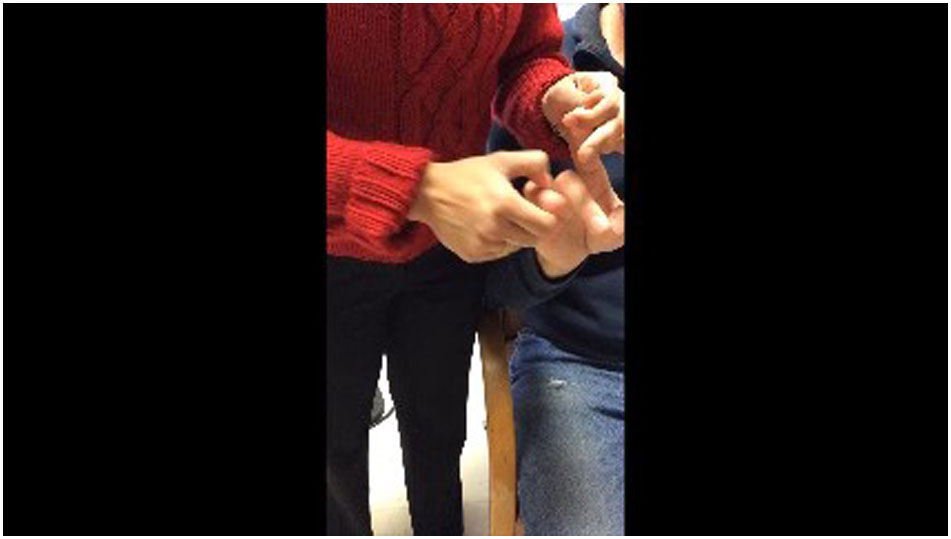

Supplement: Video 2 — Case video of wrist and finger extension before and after hyaluronidase injections. [file mmc2.jpg]

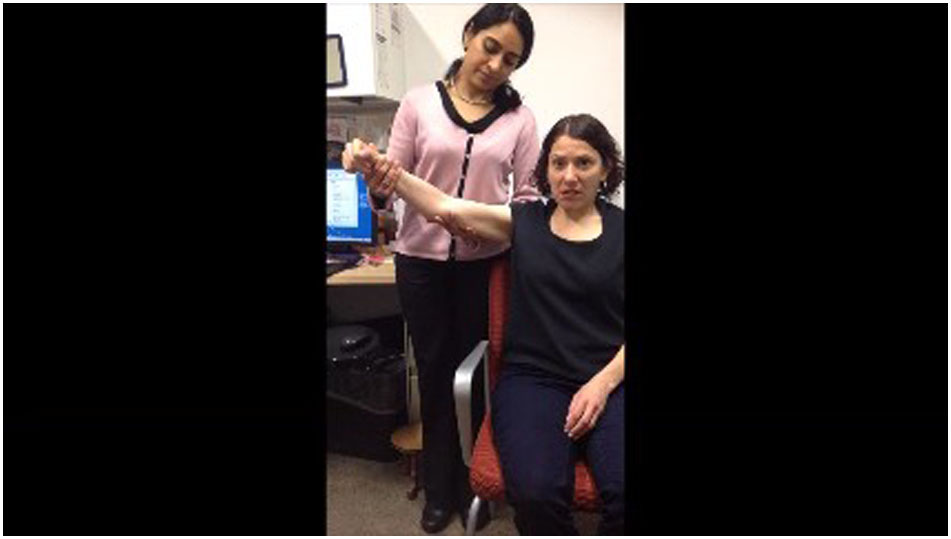

Supplement: Video 3 — Case video of passive shoulder abduction before and after hyaluronidase injections. [file mmc3.jpg]

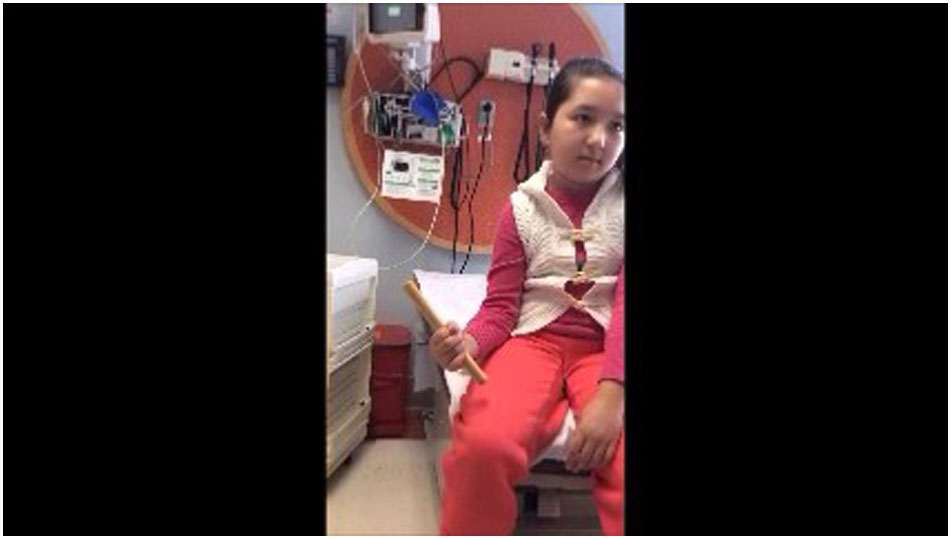

Supplement: Video 4 — Case video of active forearm pronation and supination before and after hyaluronidase injections. [file mmc4.jpg]

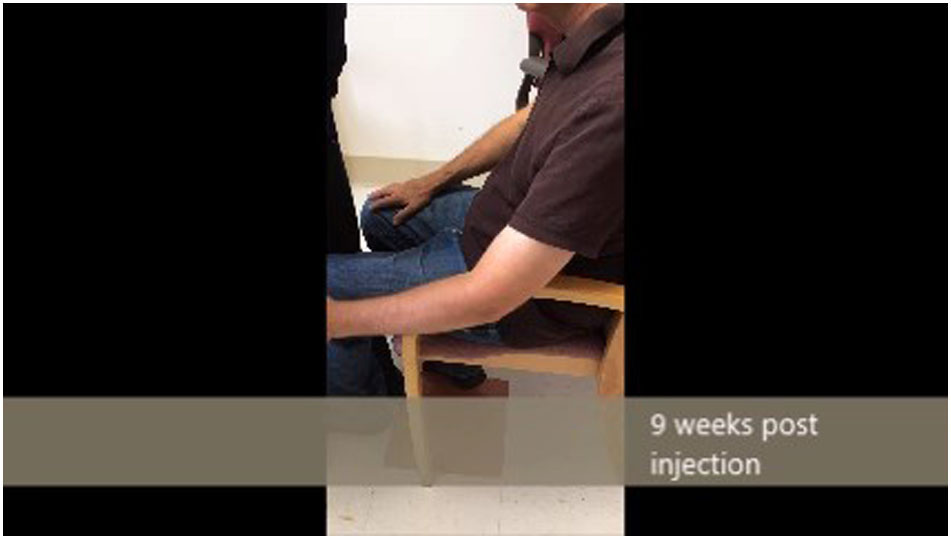

Supplement: Video 5 — Case video of passive shoulder flexion before and after hyaluronidase injections. [file mmc5.jpg]
