# Supplementary material for: Human Recombinant Hyaluronidase Injections For Upper Limb Muscle Stiffness in Individuals With Cerebral Injury: A Case Series
Source: eBioMedicine. 2016 May 13;9:306–13. doi: 10.1016/j.ebiom.2016.05.014 (PMC4972484; doi:10.1016/j.ebiom.2016.05.014)
Supplement: Appendix A — Individual subject passive and active range of motion data extracted independently by two investigators. [file mmc6.pdf]

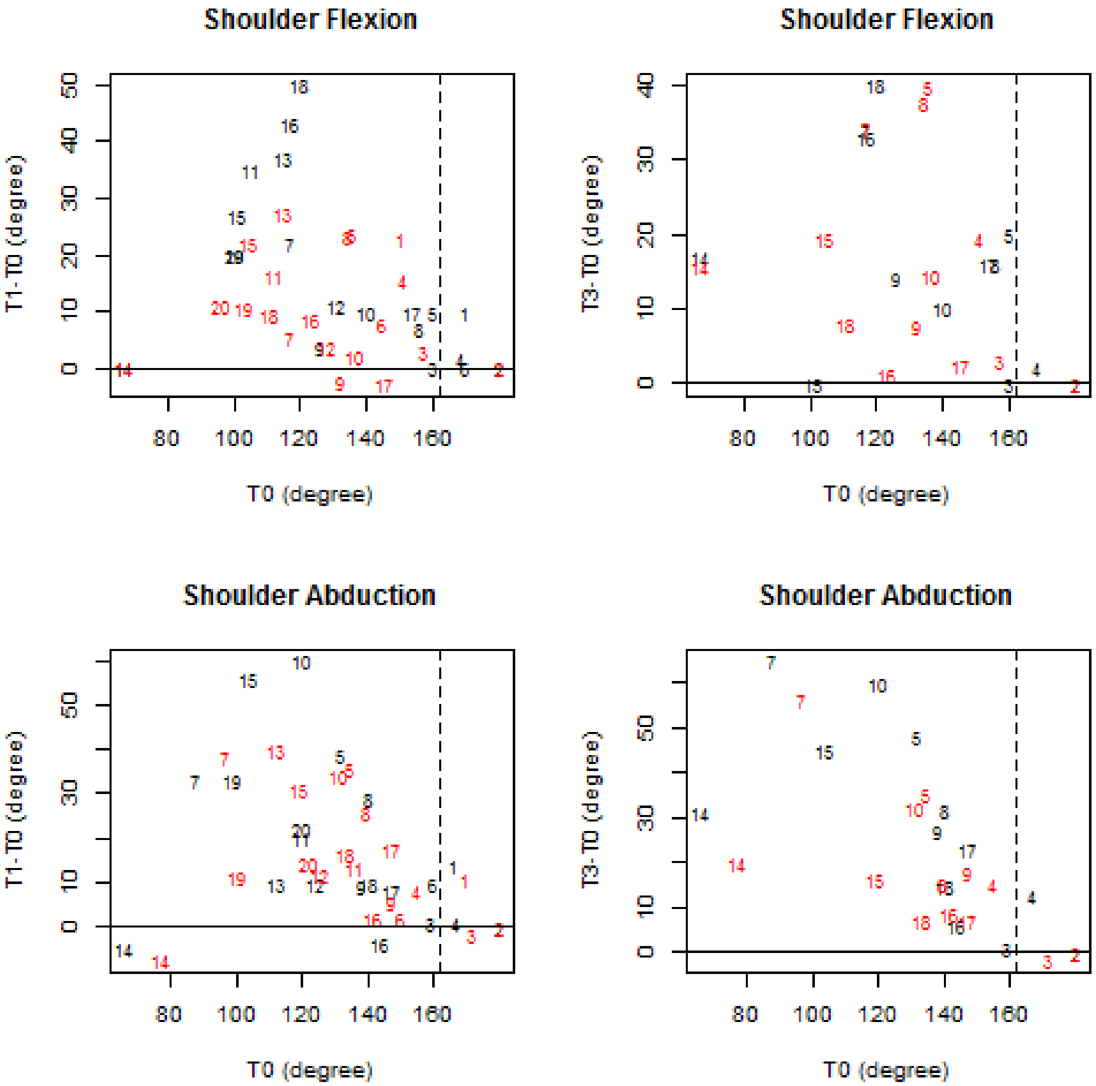

Appendix A.1: **Passive Shoulder Range of Motion**

T0=pre-injection, T1=within 2 weeks, and T3=within 3-5 months post-injection. Subjects are labelled 1-20. Back and red numbers represent values extracted independently by the two investigators. The horizontal line represents no change. The subjects above the solid horizontal line showed increase in range of motion from baseline. The dashed vertical line marks 90% of the full movement at the joint. Subjects to the right of the dashed line had within 10% of the full movement at baseline.

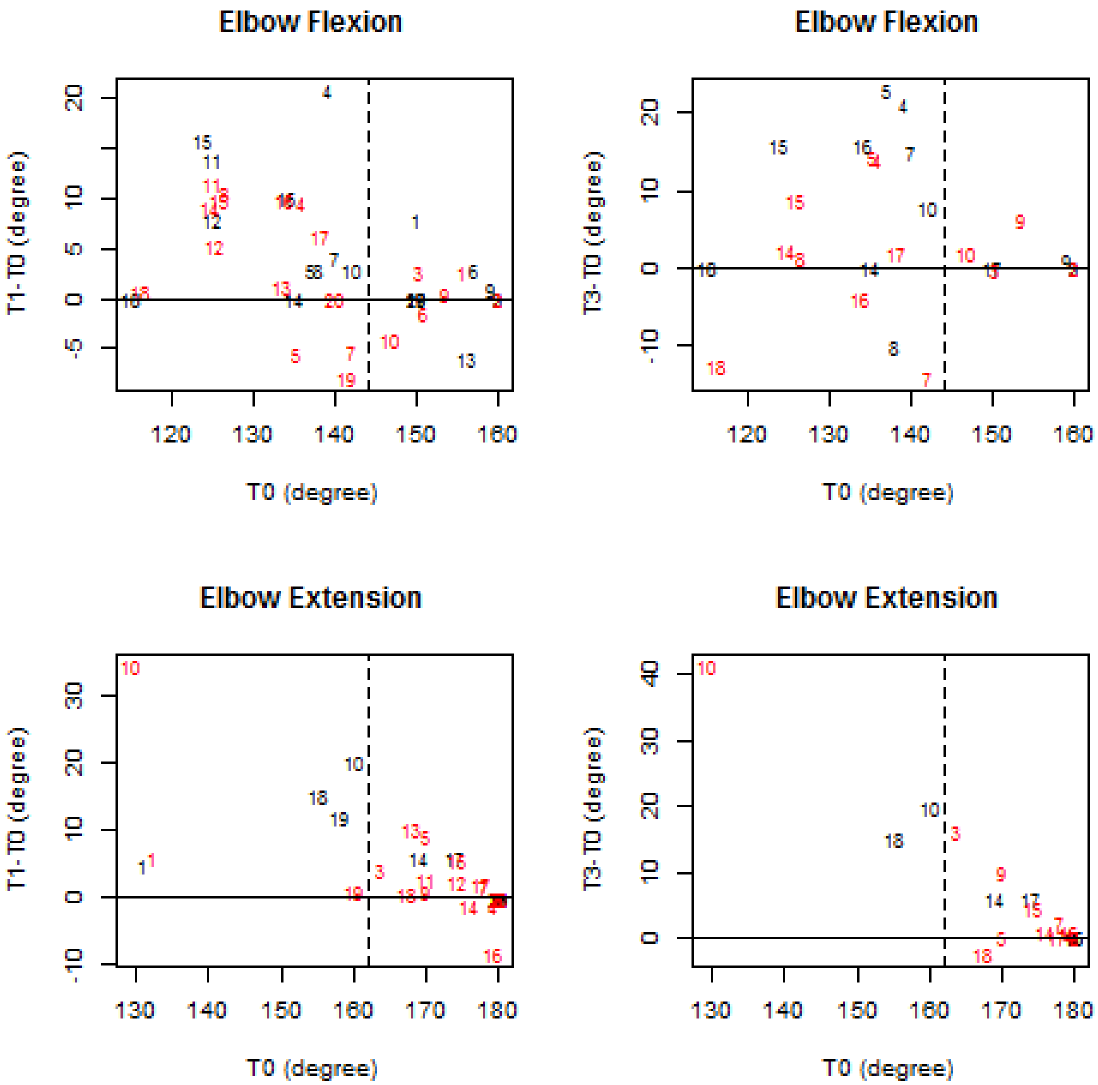

Appendix A.2: **Passive Elbow Range of Motion**
T0=pre-injection, T1=within 2 weeks, and T3=within 3-5 months post-injection. Subjects are labelled 1-20. Back and red numbers represent values extracted independently by the two investigators. The horizontal line represents no change. The subjects above the solid horizontal line showed increase in range of motion from baseline. The dashed vertical line marks 90% of the full movement at the joint. Subjects to the right of the dashed line had within 10% of the full movement at baseline.

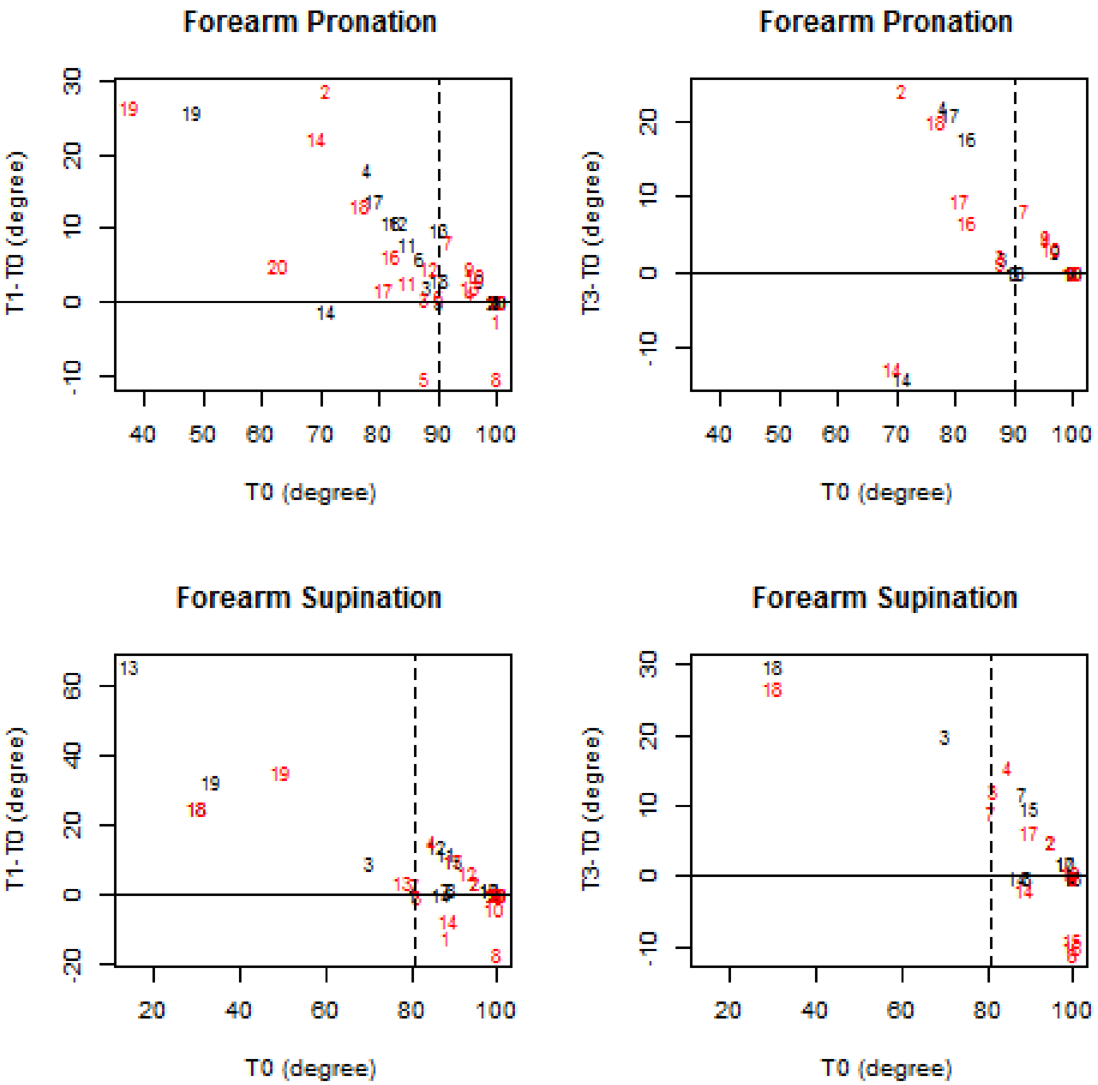

Appendix A.3: **Passive Forearm Range of Motion**
T0=pre-injection, T1=within 2 weeks, and T3=within 3-5 months post-injection. Subjects are labelled 1-20. Back and red numbers represent values extracted independently by the two investigators. The horizontal line represents no change. The subjects above the solid horizontal line showed increase in range of motion from baseline. The dashed vertical line marks 90% of the full movement at the joint. Subjects to the right of the dashed line had within 10% of the full movement at baseline.

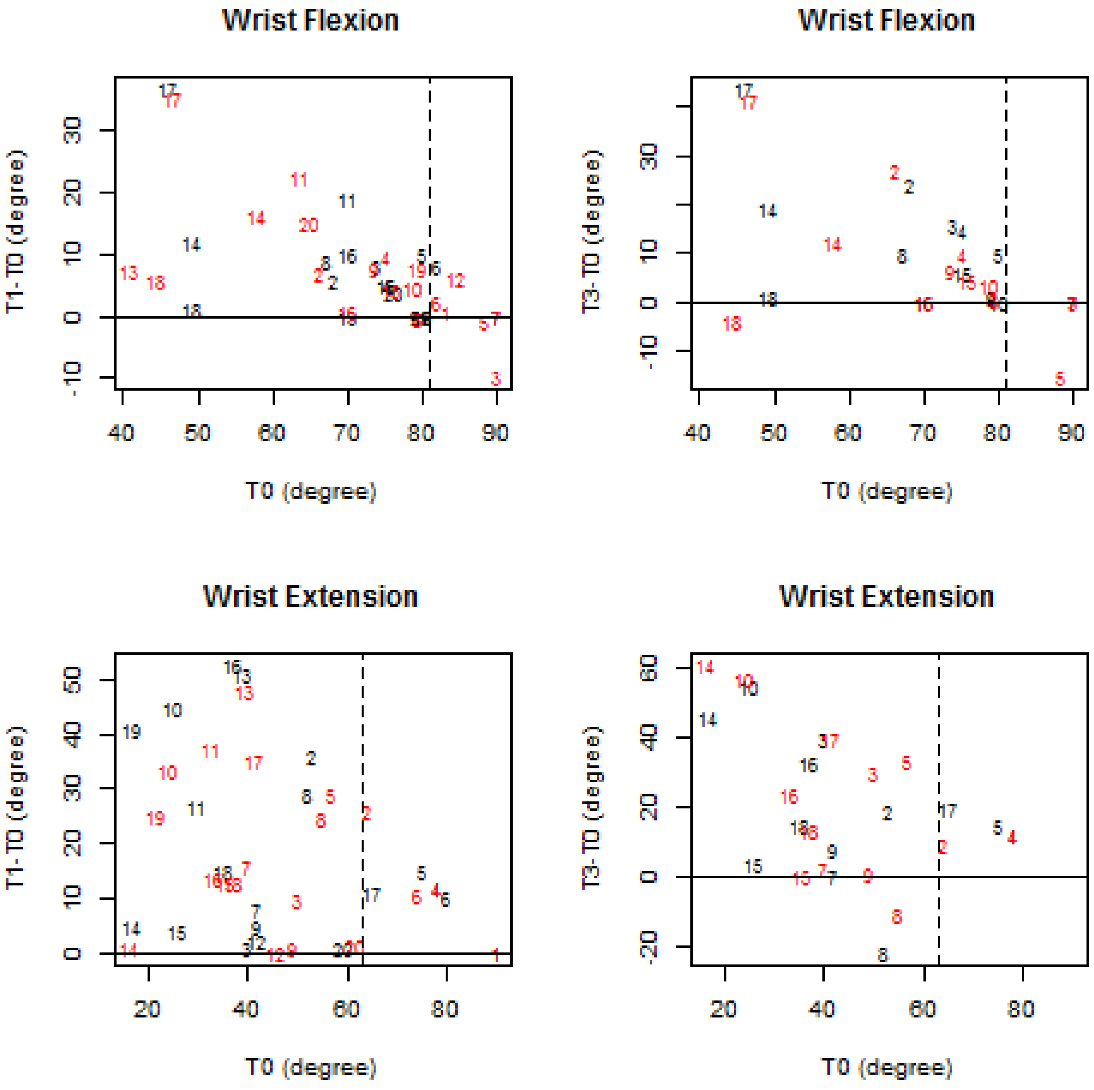

Appendix A.4: **Passive Wrist Range of Motion**
T0=pre-injection, T1=within 2 weeks, and T3=within 3-5 months post-injection. Subjects are labelled 1-20. Back and red numbers represent values extracted independently by the two investigators. The horizontal line represents no change. The subjects above the solid horizontal line showed increase in range of motion from baseline. The dashed vertical line marks 90% of the full movement at the joint. Subjects to the right of the dashed line had within 10% of the full movement at baseline.

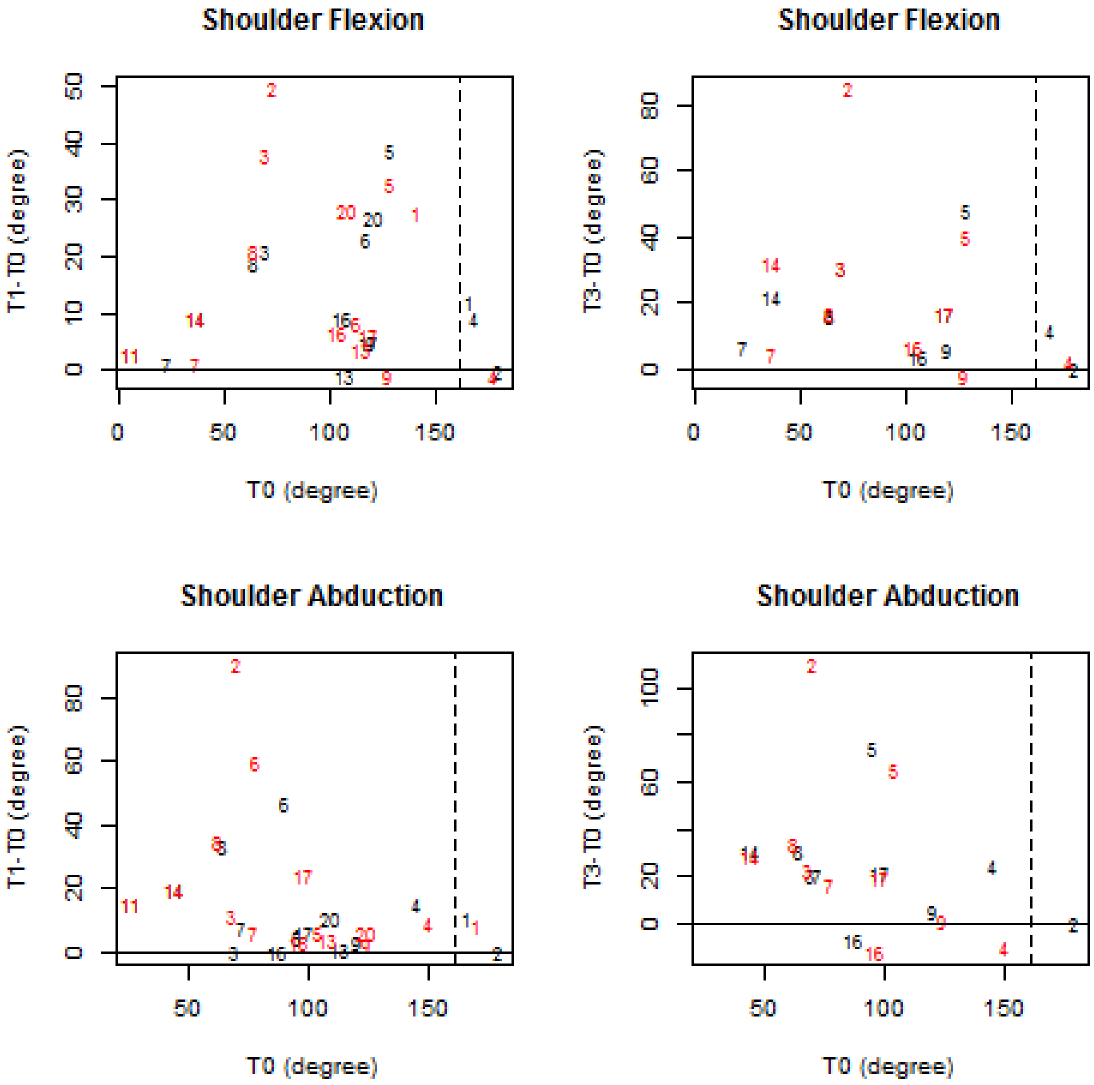

Appendix A.5: **Active Shoulder Range of Motion**

T0=pre-injection, T1=within 2 weeks, and T3=within 3-5 months post-injection. Subjects are labelled 1-20. Back and red numbers represent values extracted independently by the two investigators. The horizontal line represents no change. The subjects above the solid horizontal line showed increase in range of motion from baseline. The dashed vertical line marks 90% of the full movement at the joint. Subjects to the right of the dashed line had within 10% of the full movement at baseline.

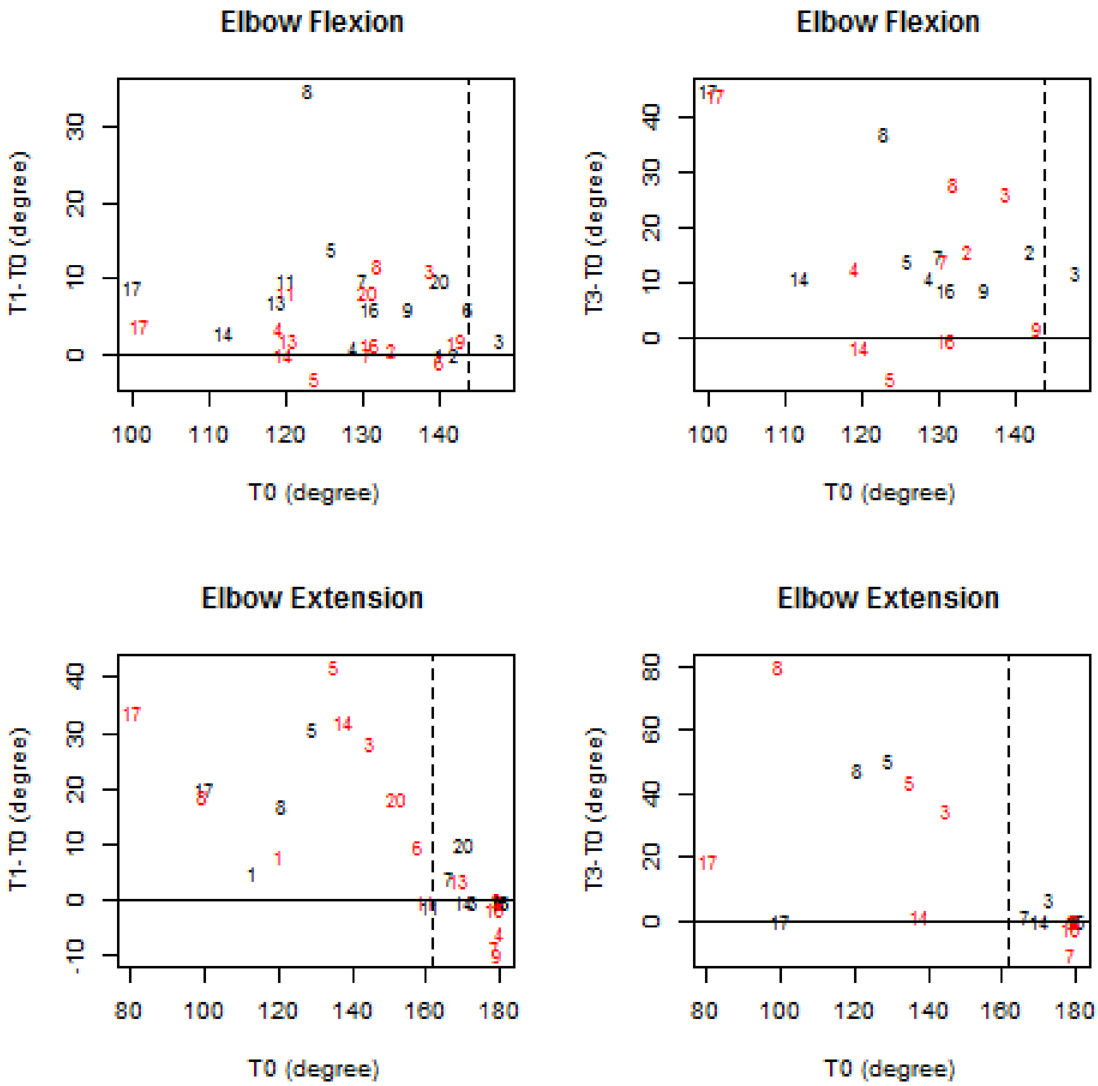

***Appendix A.6:* Active Elbow Range of Motion**

T0=pre-injection, T1=within 2 weeks, and T3=within 3-5 months post-injection. Subjects are labelled 1-20. Back and red numbers represent values extracted independently by the two investigators. The horizontal line represents no change. The subjects above the solid horizontal line showed increase in range of motion from baseline. The dashed vertical line marks 90% of the full movement at the joint. Subjects to the right of the dashed line had within 10% of the full movement at baseline.

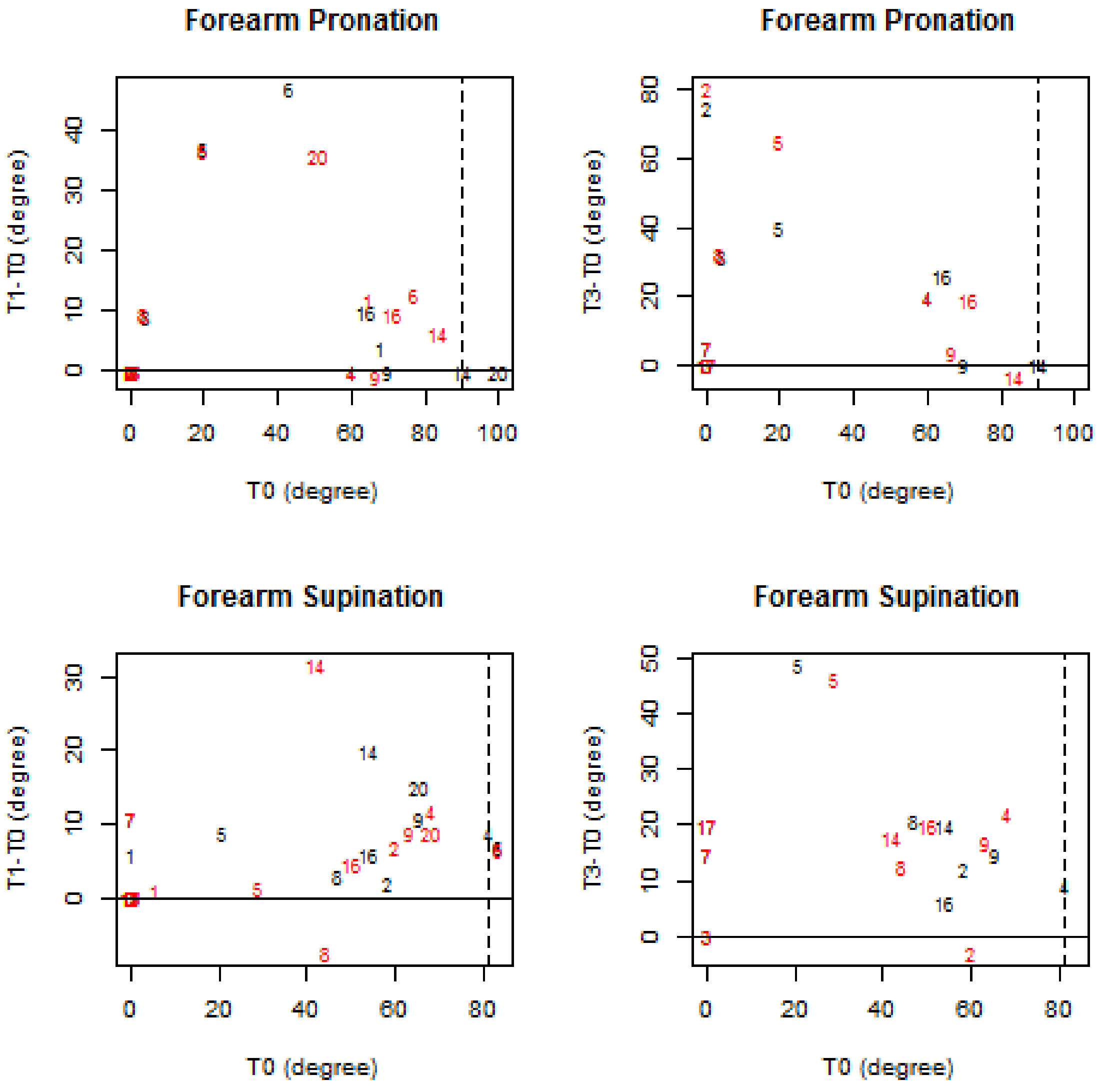

Appendix A.7: **Active Forearm Range of Motion**
T0=pre-injection, T1=within 2 weeks, and T3=within 3-5 months post-injection. Subjects are labelled 1-20. Back and red numbers represent values extracted independently by the two investigators. The horizontal line represents no change. The subjects above the solid horizontal line showed increase in range of motion from baseline. The dashed vertical line marks 90% of the full movement at the joint. Subjects to the right of the dashed line had within 10% of the full movement at baseline.

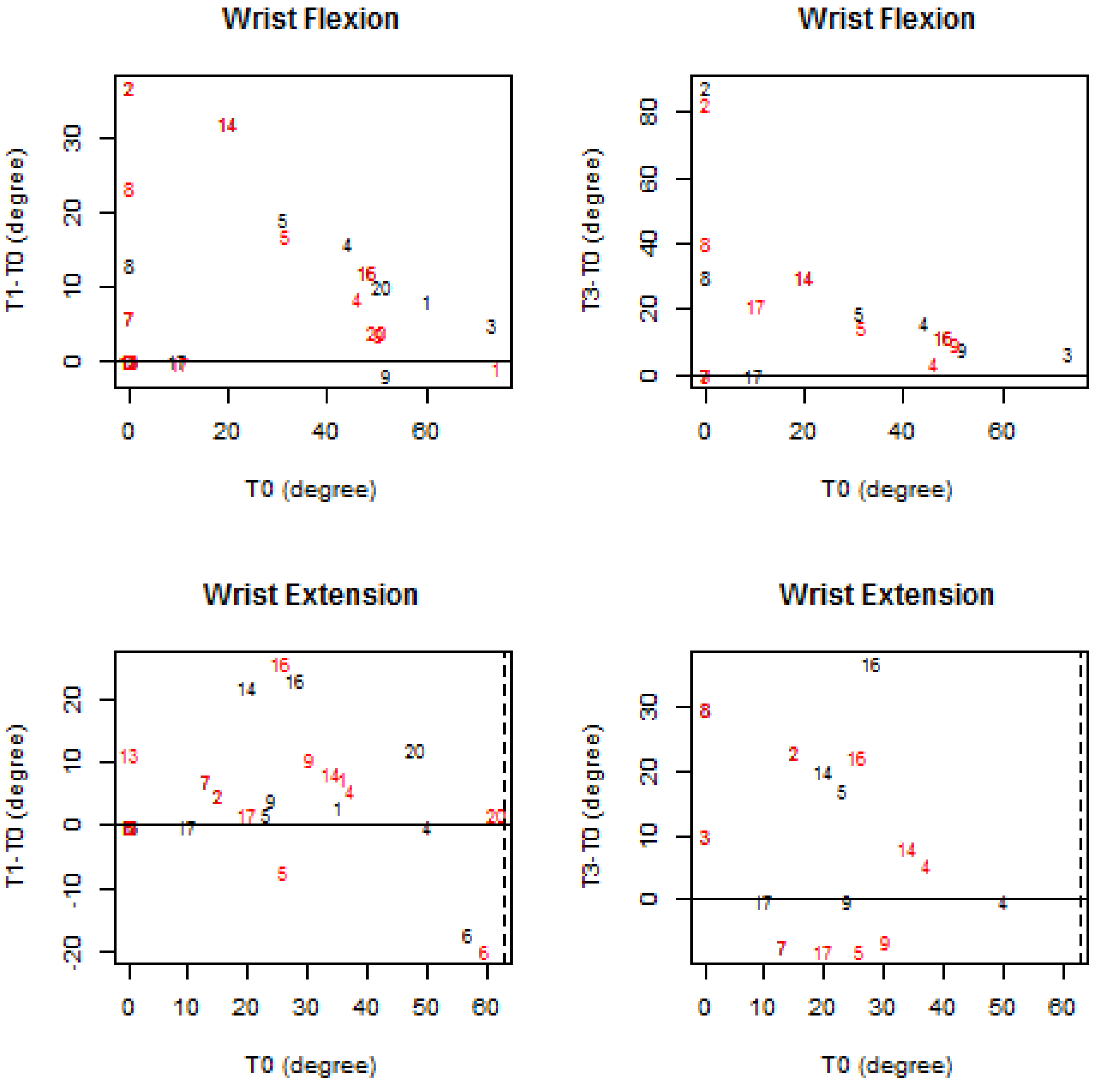

Appendix A.8: **Active Wrist Range of Motion**
T0=pre-injection, T1=within 2 weeks, and T3=within 3-5 months post-injection. Subjects are labelled 1-20. Back and red numbers represent values extracted independently by the two investigators. The horizontal line represents no change. The subjects above the solid horizontal line showed increase in range of motion from baseline. The dashed vertical line marks 90% of the full movement at the joint. Subjects to the right of the dashed line had within 10% of the full movement at baseline.
